# Two-Metal Ion-Dependent Enzymes as Potential Antiviral Targets in Human Herpesviruses

**DOI:** 10.1128/mbio.03226-21

**Published:** 2022-01-25

**Authors:** Katherine A. DiScipio, Savithri Weerasooriya, Renata Szczepaniak, Akram Hazeen, Lee R. Wright, Dennis L. Wright, Sandra K. Weller

**Affiliations:** a Department of Molecular Biology and Biophysics, University of Connecticut grid.208078.5grid.63054.34 School of Medicine, Farmington, Connecticut, USA; b Graduate Program in Molecular Biology and Biochemistry, University of Connecticut grid.208078.5grid.63054.34 School of Medicine, Farmington, Connecticut, USA; c Department of Pharmaceutical Sciences, University of Connecticut grid.208078.5grid.63054.34 School of Pharmacy, Storrs, Connecticut, USA; d Department of Chemistry, University of Connecticut grid.208078.5grid.63054.34 School of Pharmacy, Storrs, Connecticut, USA; University of North Carolina, Chapel Hill

**Keywords:** herpes simplex virus, HSV, human cytomegalovirus, HCMV, ICP8, integrase inhibitors, two-metal ion-dependent enzymes, viral nucleases, viral polymerases, cytomegalovirus, herpes alkaline nuclease, proofreading exonuclease

## Abstract

The majority of drug discovery efforts against herpesviruses have focused on nucleoside analogs that target viral DNA polymerases, agents that are associated with dose-limiting toxicity and/or a narrow spectrum of activity. We are pursuing a strategy based on targeting two-metal ion-dependent (TMID) viral enzymes. This family of enzymes consists of structurally related proteins that share common active sites containing conserved carboxylates predicted to coordinate divalent cations essential for catalysis. Compounds that target TMID enzymes, such as HIV integrase and influenza endoribonuclease, have been successfully developed for clinical use. HIV integrase inhibitors have been reported to inhibit replication of herpes simplex virus (HSV) and other herpesviruses; however, the molecular targets of their antiviral activities have not been identified. We employed a candidate-based approach utilizing several two-metal-directed chemotypes and the potential viral TMID enzymatic targets in an effort to correlate target-based activity with antiviral potency. The panel of compounds tested included integrase inhibitors, the anti-influenza agent baloxavir, three natural products previously shown to exhibit anti-HSV activity, and two 8-hydroxyquinolines (8-HQs), AK-157 and AK-166, from our in-house program. The integrase inhibitors exhibited weak overall anti-HSV-1 activity, while the 8-HQs were shown to inhibit both HSV-1 and cytomegalovirus (CMV). Target-based analysis demonstrated that none of the antiviral compounds acted by inhibiting ICP8, contradicting previous reports. On the other hand, baloxavir inhibited the proofreading exonuclease of HSV polymerase, while AK-157 and AK-166 inhibited the alkaline exonuclease UL12. In addition, AK-157 also inhibited the catalytic activity of the HSV polymerase, which provides an opportunity to potentially develop dual-targeting agents against herpesviruses.

## INTRODUCTION

The alphaherpesvirus herpes simplex virus 1 (HSV-1) has infected more than 67% of the world’s population under the age of 50 (3.7 billion people) (1), while HSV-2 infections are seeing a dramatic increase in prevalence (2). Although HSV-1 and -2 are most commonly associated with recurrent oral and genital lesions, they also cause severe ocular infections, end-stage organ disease, and life-threatening encephalitis. While infections can occur in individuals with competent immune systems, they are significantly more dangerous in immunocompromised individuals.

Currently, HSV infections are managed through prophylactic/suppressive treatment with agents that target viral DNA polymerase (Pol): nucleoside analogs (acyclovir [ACV]), nucleotide analogs (cidofovir), and pyrophosphate mimetics (foscarnet). ACV is a first-line antiviral medication used for HSV therapy and prophylaxis (3), although long-term use can lead to the development of drug resistance, especially in immunocompromised patients (4–7). Viral shedding is frequently observed in immunocompetent individuals treated with ACV, suggesting that elimination or complete suppression of the virus cannot be achieved with the current treatment options (8, 9). HSV-1 and -2 are members of the larger family of human herpesviruses (HHVs) that include pathogens such as cytomegalovirus (CMV), Kaposi’s sarcoma virus (HHV-8), and the roseola viruses (HHV-6A/6B/7), which are associated with significant morbidity and mortality. ACV is ineffective against these other HHVs; however, these viruses can be treated with the nucleoside analog ganciclovir (GCV). Unfortunately, however, GCV is associated with severe nephrotoxicity and myelosuppression, which can force discontinuation of therapy (10). Furthermore, prolonged prophylactic use of GCV is also associated with the onset of antiviral drug resistance (11). New therapies with novel modes of action would be important not only for the treatment of resistant viruses but also for use in combination therapy to lower dose-limiting toxicities to prevent the emergence of resistance. Indeed, such combinations could prove useful in preventing the spread of HHVs. Since many essential HHV proteins are well conserved, inhibitors of these novel targets could exhibit broad-spectrum activity against multiple HHVs.

Approximately half of the 80 to 90 open reading frames (ORFs) in the HSV genome encode essential proteins. The majority of those essential functions are performed by enzymes involved in the replication and processing of viral DNA, particularly the seven encoded essential DNA replication proteins: polymerase (UL30), polymerase accessory factor (UL42), the three-subunit helicase/primase (UL5/UL52/UL8), origin binding protein (UL9), and the single-stranded DNA (ssDNA) binding protein (ICP8) (12). In addition, HSV encodes a 5′-to-3′ alkaline exonuclease, UL12, which is essential for the production of viral DNA that can be packaged into infectious virus (13–16). The products of HHV DNA replication are longer-than-unit-length genomes (concatemers) that are cleaved by the viral terminase (UL15/UL28/UL33) prior to encapsidation (17–19). The largest subunit of the complex, UL15, is responsible for the endonucleolytic cleavage event. The critical nature of these enzymes provides an opportunity to establish novel targets for drug discovery. Both UL12 and UL15 have been shown to be essential for viral replication, and some exploratory efforts to identify inhibitors of these enzymes have been undertaken (16, 20–25). Additionally, the CMV-specific inhibitor letermovir, which binds to an allosteric site on the CMV terminase (UL89), has been recently approved (26–28).

Nucleic acid processing proteins are vital to the replication of all viruses. Structurally, many of these enzymes fit into a large class of two-metal ion-dependent (TMID) enzymes, whose functional roles include RNA and DNA digestion, DNA integration, and recombination. The active sites of TMID enzymes typically contain 3 to 4 conserved acidic residues (DDE or DEDD motifs), which coordinate two divalent cations such as magnesium. The tandem metals are essential for substrate binding and subsequent cleavage of the phosphodiester bond (29, 30). The TMID proteins are divided into different superfamilies, including the RNase H-like (RNHL) superfamily (29, 31, 32), PD(D/E)XK (shortened as DEK) motif proteins, in which three acidic residues are located close to each other on a β-hairpin (30), and the B-family DNA polymerases, which contain a proofreading exonuclease domain (33). HSV encodes several bona fide TMID enzymes from different superfamilies: the UL12 alkaline nuclease (DEK family) (34), the UL15 terminase (RNHL family) (35), and UL30, a B-family polymerase with a 3′-to-5′ exonuclease domain, PolExo (36–40), which is also classified as an RNHL family member (29). Interestingly, Bryant et al. reported the identification of a divalent metal cation binding site in HSV ICP8 (41) and suggested that ICP8 could be a two-metal binding protein, although it does not bear obvious structural homology to any of the well-described TMID superfamilies.

Drug discovery efforts against TMID proteins in HIV and influenza have resulted in the development of selective and safe inhibitors, illustrating that enzymes of this class can be viable drug targets (32, 42–44). HIV integrase is a classic RHNL family member (29, 30), while the influenza endoribonuclease (the PA subunit of the viral polymerase) is a member of the DEK family (45–47). Several reports indicate that HIV integrase inhibitors can inhibit replication of HSV, CMV, Kaposi sarcoma-associated herpesvirus, and feline herpesvirus 1 (23, 48, 49); however, the molecular targets of their antiviral activities have not been definitively identified. It has been suggested that integrase inhibitors act by blocking the function of ICP8 and/or UL15 (23, 48, 49). In this article, we utilized a candidate-based approach involving multiple two-metal-directed compounds and several viral enzymatic targets in order to correlate target-based activity with antiviral potency.

## RESULTS

Known inhibitors of TMID enzymes typically have a planar array of three adjacent heteroatoms (N or O) that can associate simultaneously with both Mg^2+^ atoms in the target protein. We assembled a panel of known and potential TMID inhibitors to screen against the potential targets in HHVs. The panel consisted of two integrase inhibitors with reported anti-HSV activity (raltegravir [RAL] and the experimental agent XZ45), three newer integrase inhibitors (dolutegravir [DTG], MK-2048, and cabotegravir [CAB]), and a recently approved influenza antiviral agent that targets the endonuclease (cap-snatching) function of the PA subunit of the viral RNA-dependent RNA polymerase (RdRp), baloxavir acid (BXA) (50). We hoped to augment this panel with commercially available leads predicted to interact with two-metal active sites; however, the available large libraries of small molecules contained surprisingly few compounds possessing the requisite array. We also chose to include two naturally occurring tropolone derivatives (purpurogallin [PPG] and β-thujaplicinol [BTP]). BTP was initially identified in a screen for inhibition of HIV RNase H activity (51), and both compounds were previously shown to have potent anti-HSV activity (52). We further supplemented this panel with two 8-hydroxyquinolones (8-HQs), AK-157 and AK-166, from our in-house TMID inhibitor library as this chemotype has previously been shown to display anti-HSV activity (53). Our primary goal was to measure the relative antiviral activity of compounds that possess similar, but not identical, metal-directed pharmacophores and correlate that activity with target-level inhibition of the possible enzyme targets.

### Inhibition of HSV replication.

**FIG 1:**
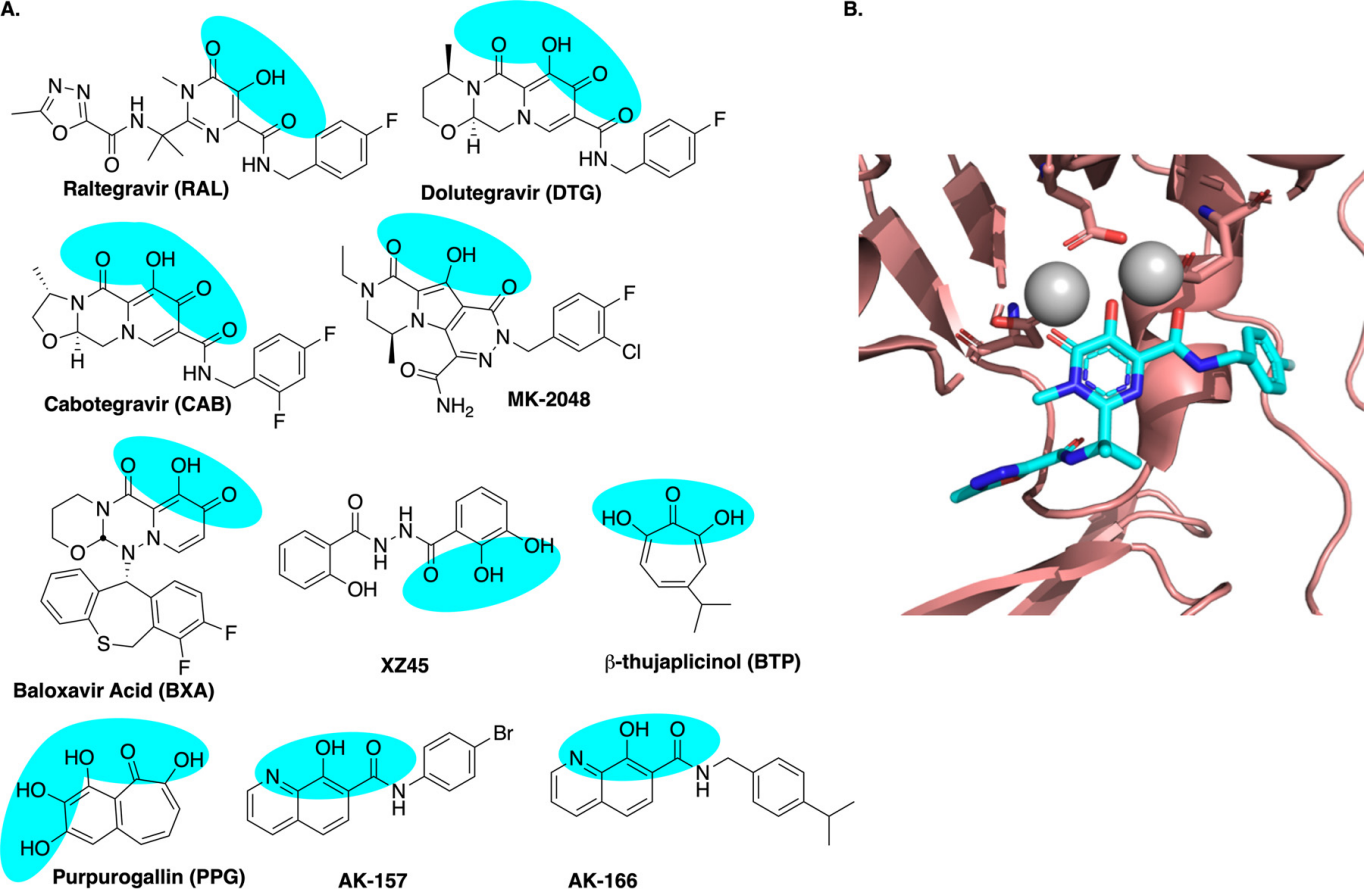
Small molecule agents targeting two-metal ion-dependent enzymes (A) Blue highlighted regions indicate planar arrays of heteroatoms that can form two or more adjacent chelates. (B) Structure of raltegravir bound to the prototype foamy virus (PFV) intasome showing the coordination of drug to the two catalytic magnesium ions that are bound to the enzyme through a DDE motif (PDB ID no. 3OYA).

**FIG 2:**
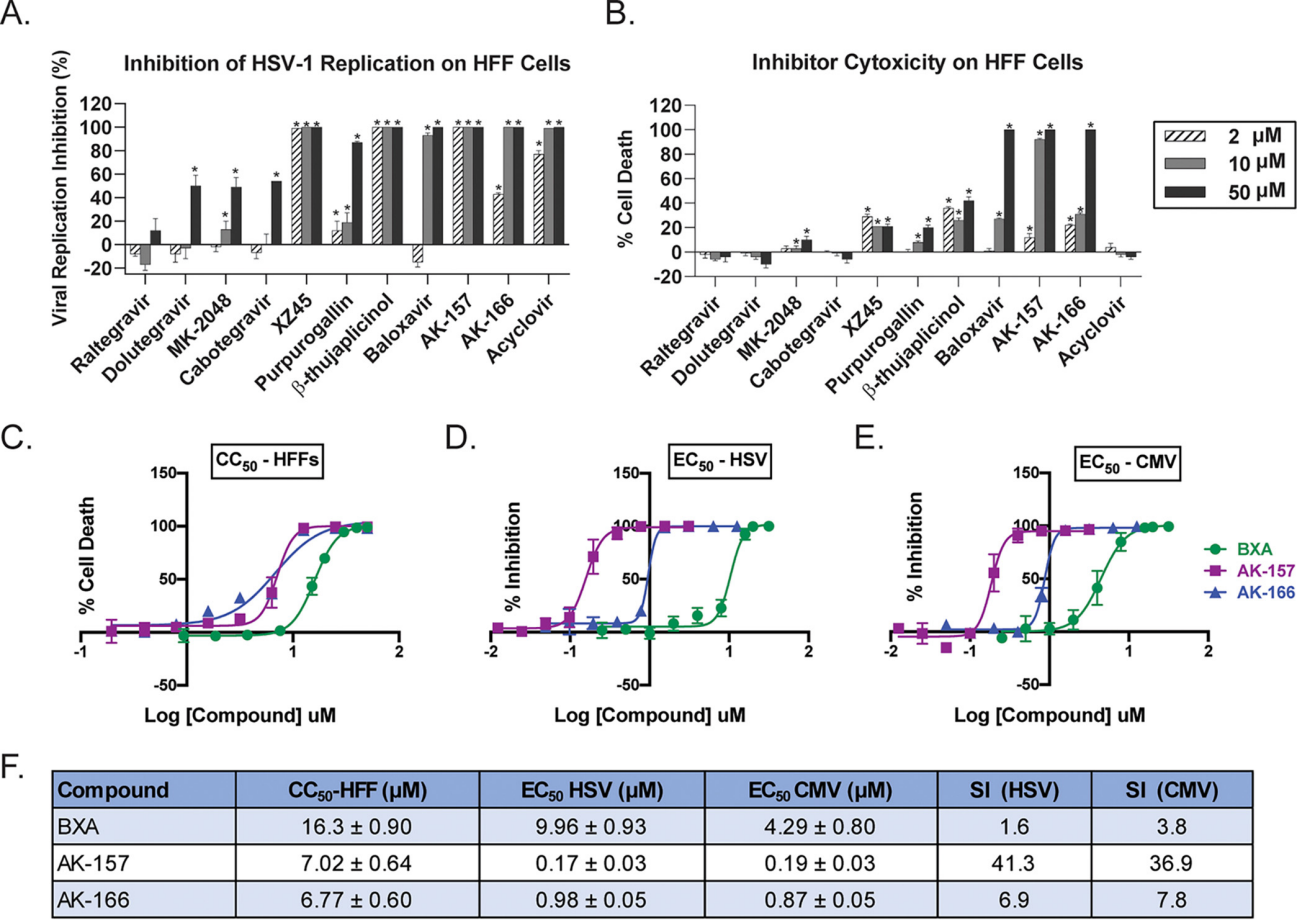
Antiviral activity and cytotoxicity of compounds used in this study. (A) HFF cells were infected with KOS at an MOI of 0.1 PFU/cell in the presence of compound at concentrations of 2, 10, and 50 μM. At 48 hpi, cultures were collected, and viral titer was determined by plaque assay. The percentage of inhibition was calculated compared to the DMSO control. Values are the averages of data from three independent experiments. (B) Cytotoxicity of compounds on HFF cells after a 48-h incubation period was evaluated using the Promega Cell Titer-Glo assay, following the manufacturer’s instructions. (C) Dose-dependent cytotoxicity of BXA, AK-157, and AK-166 on HFF cells at 65 h posttreatment was measured using Promega Cell Titer-Glo. (D) HFF cells were infected with HSV (KOS strain) at an MOI of 0.1 PFU/cell, and viral titers were determined by plaque assay at 48 hpi. (E) HFF cells were infected with HCMV (Towne strain expressing pp28-luciferase) at an MOI of 2 PFU/cell, and viral infection was monitored at 65 hpi using Promega SteadyGlo. The data shown in panels D and E represent three duplicates within one experiment. (F) Calculated EC_50_ and 50% cytotoxic concentration (CC_50_) values for BXA, AK-157, and AK166 represent averages from three independent experiments. The selectivity index (SI) is given as CC_50_/EC_50_. *, *P* < 0.05 compared with the DMSO control by two-way ANOVA and Holm-Sidak multiple-comparison test. CC_50_ and EC_50_ values were compared with a nonlinear regression model, and groups were significantly different at *P* < 0.05.

The agents shown in Fig. 1 were tested for inhibition of HSV-1 viral replication at three concentrations (2, 10, and 50 μM) in human foreskin fibroblasts (HFFs) using ACV as a positive control. Although some previous studies tested integrase inhibitors at higher concentrations, we selected 50 μM as our maximum as this concentration likely exceeds achievable serum levels (54, 55). Figure 2A shows that ACV inhibited HSV at all concentrations. While Zhou et al. reported an 50% effective concentration (EC_50_) value of 67.7 μM for inhibition of HSV-1 by RAL (49), in our hands the drug showed no signs of antiviral activity, even at 50 μM. The newer-generation integrase inhibitors (DTG, CAB, and MK-2048), however, showed moderate inhibition, with approximately 50% reduction in viral yields at 50 μM. None of the integrase inhibitors, except perhaps MK-2048, were cytotoxic at the concentrations tested (Fig. 2B). Only the experimental integrase inhibitor XZ45 showed relatively strong anti-HSV activity at all three concentrations, although with an increase in cytotoxicity relative to the approved integrase inhibitors. This result is consistent with a previous report showing that XZ45 is more potent against HSV-1 than the clinically used integrase inhibitors such as RAL and DTG (48).

The PA endoribonuclease inhibitor BXA showed stronger antiviral activity and higher toxicity than the integrase inhibitors. Notably, complete inhibition was achieved at 10 μM BXA (Fig. 2A). As previously reported (25), the naturally occurring tropolones showed anti-HSV activity, with BTP associated with higher antiviral and cytotoxicity levels relative to PPG. The two 8-HQs (AK-157 and AK-166) showed strong anti-HSV activity even at the 2 μM concentration, with cytotoxicity similar to that of XZ45; however, at the higher concentrations, more significant cytotoxicity was observed. Taken together, 5 of the 10 tested compounds (BTP, XZ45, BXA, AK-157, and AK-166) showed significant antiviral activity between 2 and 10 μM; of these, only the anti-HSV activities of BTP and XZ45 have been previously reported (48, 52).

Dose-response experiments for HSV-1 and CMV were conducted with BXA, AK-157, and AK-166 (Fig. 2C). HFF cells were infected with KOS or the pp28-luciferase-expressing human cytomegalovirus (HCMV) Towne strain and incubated for 48 or 65 h, respectively, in the presence of various concentrations of compounds. For the HSV inhibition assay, viral yields were assessed by plaque assay. For CMV inhibition, cells were monitored for luciferase expression. It should be noted that because the antiviral activities are determined at different stages of the viral life cycle, it may not be appropriate to directly compare EC_50_ values between HSV and CMV activities. However, these experiments do allow us to rank order compound potency for a given virus. BXA showed modest HSV-1 and CMV antiviral activity (EC_50_ values of 9.96 and 4.29 μM, respectively). However, BXA also shows relatively high levels of cytotoxicity, resulting in very narrow selectivity indices (SIs). The 8-HQ derivatives AK-157 and AK-166 were substantially more potent than BXA, with AK-157 being superior (EC_50_ values of 0.17 μM for HSV-1 and 0.19 μM for CMV). AK-157 displayed reduced levels of cytotoxicity compared to BXA, leading to overall higher SI values of 41.3 and 36.9 for HSV-1 and CMV, respectively.

### Biochemical studies of TMID inhibition by small molecules.

In parallel, we conducted an unbiased, biochemical candidate-based approach to determine key structure-activity relationships for inhibition of the TMID target enzymes. Previous reports suggested ICP8 and UL15, two essential HSV-1 proteins, as potential targets of integrase inhibitors, providing a logical starting point for screening.

### The annealing function of ICP8 is not inhibited by integrase inhibitors.

ICP8 catalyzes the annealing of complementary ssDNA in a Mg^2+^-dependent manner, similar to the Rad52 and Redβ annealing proteins (56–58). ICP8 has, consequently, been classified as a member of the ssDNA annealing protein (SSAP) family (59). Based on the presence of a putative divalent metal cation binding site in ICP8, as well as reported phenotypic effects on viral replication and recombination (41, 48), ICP8 has been suggested to be a possible target of RAL and XZ45. We have recently demonstrated that the annealing activity of ICP8 is essential for HSV-1 viral DNA replication, consistent with the notion that HSV-1 uses recombination-dependent mechanisms to replicate its DNA (60). Thus, we were eager to determine whether integrase inhibitors were exerting their antiviral effects by blocking this essential function.

**FIG 3:**
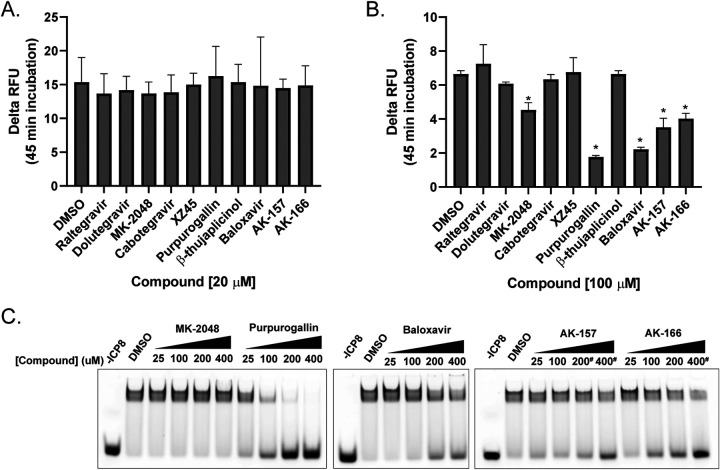
Inhibition of ICP8-mediated activities. ICP8 (100 nM) was preincubated with 20 μM (A) or 100 μM (B) compound in 1.6% DMSO for 10 min at 37°C. Annealing reactions were initiated by addition of 50 ng linearized, heat-denatured plasmid DNA, and the mixtures were incubated at 37°C for 45 min. The presence of dsDNA was measured by PicoGreen fluorescence, and the data represent the average from three independent experiments. (C) ssDNA binding by ICP8 (200 nM) was assessed by EMSA in the presence of select compounds at the concentrations shown using 100 nM Cy5-labled 25-nt dT oligomer. Binding reactions (30 min at 37°C) were analyzed by 5% nondenaturing polyacrylamide gel electrophoresis. For panels A and B, the data represent the average from three independent experiments. *, *P* < 0.05 compared with DMSO control by one-way ANOVA and Holm-Sidak multiple comparisons test. For panel C, representative data from three independent experiments are shown. *, *P* < 0.05 compared with the DMSO control by one-way ANOVA and Holm-Sidak multiple-comparison test. # indicates that at these concentrations, compound precipitation was observed.

In order to correlate antiviral activity with inhibition of ICP8 function, we assessed the ability of the compounds to prevent annealing of complementary ssDNA (linearized, heat-denatured plasmid DNA) at 20 μM (Fig. 3A). This concentration, which is approximately 3-fold lower than the reported anti-HSV EC_50_ of RAL (67 μM), was selected because target-level inhibition typically exceeds antiviral activity when a single target is inhibited. In the negative control, no annealing was observed after 45 min of incubation in the absence of ICP8 (data not shown). In the positive control (dimethyl sulfoxide [DMSO]), maximum annealing was observed by 45 min in the presence of ICP8 (Fig. 3A). Strikingly, at a 20 μM inhibitor concentration, there was no evidence of inhibition of annealing by any of the compounds. As a result of this observation, we decided to evaluate the compounds at concentrations significantly above their EC_50_ to allow detection of even weak ICP8 inhibition. At 100 μM, four or five of the compounds (PPG, BXA, AK-157, AK-166, and perhaps MK-2048) appeared to slightly reduce the annealing efficiency of ICP8 (Fig. 3B). Using these compounds, we also performed an electrophoretic mobility shift assay (EMSA) with an ssDNA substrate (Cy5-labeled 25-mer dT oligomer) to determine whether inhibition was related to ssDNA binding. BXA, AK-157, and AK-166 displayed a slight disruption of ssDNA binding starting at 100 μM (Fig. 3C). For these three compounds, the effects on ICP8 are seen at substantially higher concentrations than the antiviral activity reported above, suggesting that inhibition of ICP8 is likely not a significant component of their antiviral effects. On the other hand, PPG showed a more significant decrease in ssDNA binding starting at 25 μM. It is noteworthy that PPG was previously shown to disrupt the interaction between ssDNA and other ssDNA binding proteins, including bacterial SSB and replication protein A (RPA) (61), indicating that this compound is a nonselective disrupter of protein-DNA interactions, possibly through the extrication of metals from the active sites. A target engagement experiment (62) was subsequently conducted to confirm our suspicions about PPG. The thermal shift assay was used to measure the thermal stability of a preformed complex of ICP8 with either RAL or PPG. Analysis indicated that RAL had no appreciable interactions with ICP8, while PPG showed a modest effect (see Fig. S1 in the supplemental material). These values were unchanged by the addition of ssDNA to the preformed complex, indicating that there was no significant DNA-dependent binding. Of the integrase inhibitors, only MK-2048 had any effect on annealing. That effect was, however, relatively small and occurred in a DNA-independent manner. Overall, these results suggested that the compounds in our panel did not directly inhibit the annealing function of ICP8, prompting us to investigate the other potential TMID targets in HSV.

10.1128/mbio.03226-21.1 FIG S1 Characterization data for compounds AK-157, AK-166 and XZ45 synthesized in this study. (A) ^1^H NMR spectra for AK-157. (B) ^13^C NMR spectra for AK-157. (C) ^1^H NMR spectra for AK-166. (D) ^13^C NMR spectra for AK-166. Download FIG S1, PDF file, 0.8 MB. Copyright © 2022 DiScipio et al. 2022 DiScipio et al. https://creativecommons.org/licenses/by/4.0/ This content is distributed under the terms of the Creative Commons Attribution 4.0 International license.

### UL15 terminase activity is broadly sensitive to metal-binding pharmacophores.

**FIG 4:**
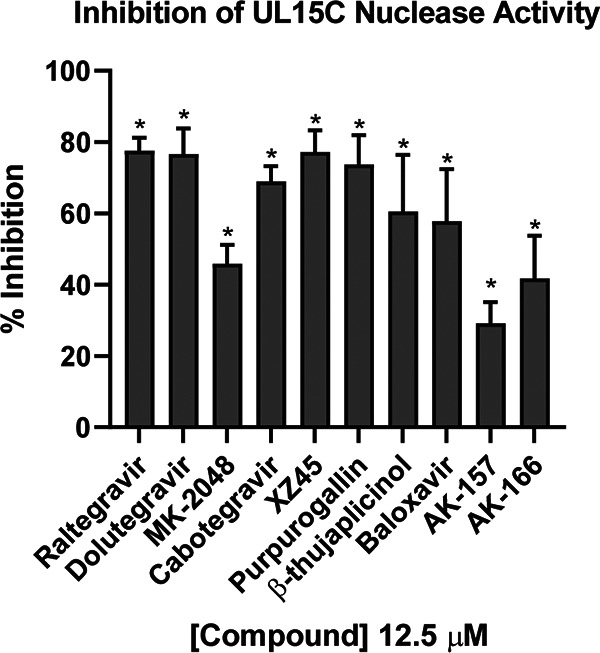
Inhibition of UL15C nuclease activity. A dual-probe assay was used to access inhibition of UL15C nuclease activity at 12.5 μM. The percentage of inhibition due to compounds was calculated compared to that of the uninhibited DMSO control. The data shown represent an average from three independent experiments in which each experiment is an average of three technical replicates. *, *P* < 0.05 compared with the DMSO control by one-way ANOVA and Holm-Sidak multiple-comparison test.

The X-ray structure of the C-terminal domain of UL15 reveals an RNase H-like fold with a cluster of acidic residues within the active site (35). UL15 has previously been investigated as a potential target for both the integrase inhibitors (23) as well as the catechol and tropolone natural products (21). We utilized a fluorescent reporter assay developed by Masaoka et al. to monitor the non-sequence-specific Mg^2+^-dependent nuclease activity of the UL15 C-terminal catalytic domain (21) to compare inhibitory activities across the entire panel of compounds (Fig. 4). The agents were tested at a fixed concentration of 12.5 μM, and all 10 compounds showed significant inhibitory activity, ranging from 25 to 80%. Because of the strong inhibition of UL15, testing at higher concentrations was not necessary. There was, however, no apparent correlation between the strength of inhibition of UL15C and the observed antiviral activity in cell culture (Fig. 2). For example, the strongly antiviral 8-HQs showed the lowest levels of UL15C inhibition, while the weakly antiviral integrase inhibitors showed the strongest activity. While we cannot rule out that inhibition of the terminase function could contribute to the overall antiviral activity of the compounds, the poor correlation suggests that the antiviral activity was not predominantly mediated by UL15 inhibition.

### UL12 alkaline nuclease is sensitive to 8-HQs and BXA, but not integrase inhibitors.

**FIG 5:**
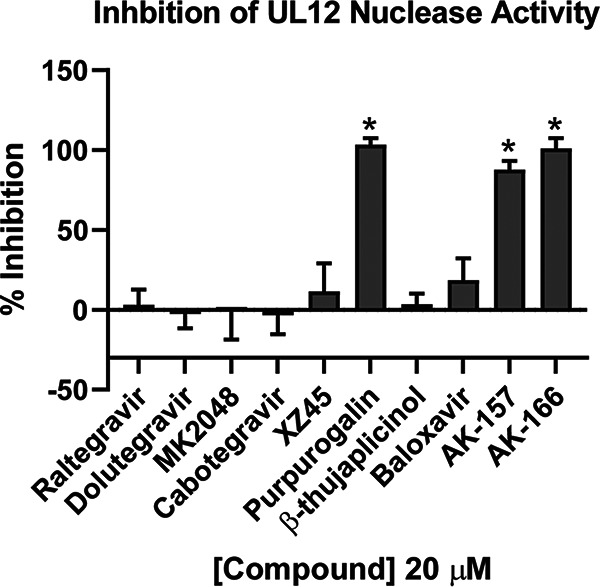
Inhibition of UL12 nuclease activity. UL12 nuclease activity was measured using the PicoGreen assay at a 20 μM inhibitor concentration. Any background fluorescence signals derived from inhibitors were subtracted from corresponding total RFU values, and the percentage of inhibition of UL12 nuclease activity was calculated compared to that of the uninhibited DMSO control. Data presented represent an average from three independent experiments in which each experiment is an average of three technical replicates. *, *P* < 0.05 compared with the DMSO control by one-way ANOVA and Holm-Sidak multiple-comparison test.

The HHV alkaline nucleases are well conserved across the herpesvirus family and have been classified as DEK nucleases based on sequence/structure threading, molecular modeling (34), and genetic analysis (15, 16). UL12 exonuclease activity was monitored by reduction of fluorescence of PicoGreen, a double-stranded DNA (dsDNA)-binding fluorochrome (16, 63). In contrast to UL15, there was a direct correlation between antiviral activity and inhibition of UL12 activity. At a fixed concentration of 20 μM, strong inhibition was observed for PPG, AK-157, and AK-166 (Fig. 5). Although XZ45 and BTP are strongly antiviral, they exhibited little inhibitory activity against UL12. These data suggest that AK-157 and AK-166 act, at least in part, through inhibition of UL12, a result consistent with our previous findings that an exonuclease-inactivating mutation (D340E) introduced into the viral genome showed the same growth defects as a UL12 null mutant (16).

### BXA and 8-HQs target different functions of UL30.

**FIG 6:**
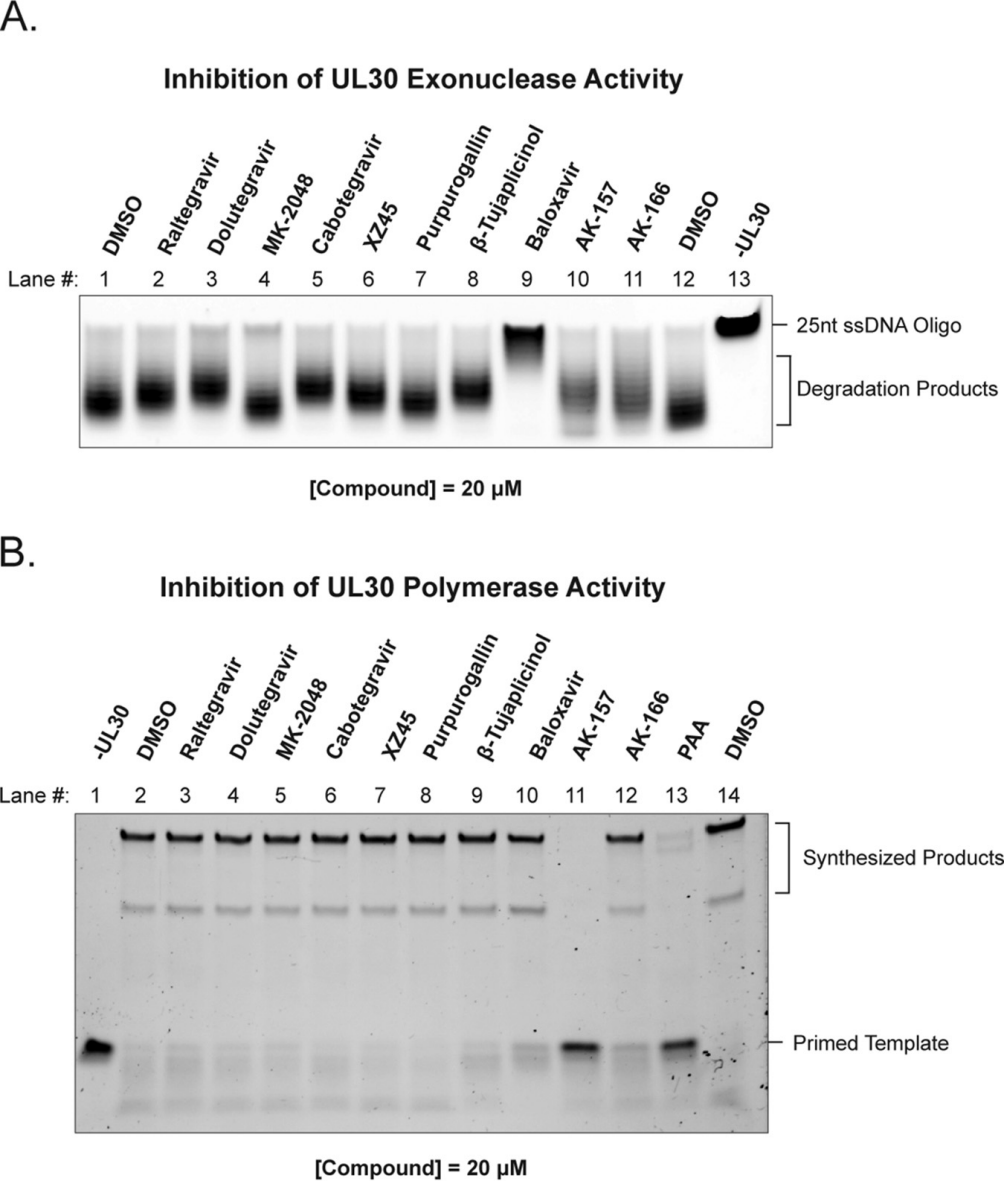
Inhibition of UL30 functions. (A) PolExo activity was measured using a gel-based assay to detect degradation of 5′-Cy5-tagged 25-mer oligo(dT) substrate using full-length His-tagged UL30 at a 20 μM drug concentration. (B) Polymerase activity was assayed using an N-terminally-deleted version of UL30 (UL30ΔN42) at a 20 μM drug concentration. A gel-based assay was used to monitor extension of a primer/template consisting of a 5′-FAM-labeled 15-nt primer annealed to a 35-nt oligonucleotide. The data shown are representative of three independent experiments, and representative panels are shown.

In addition to the C-terminal catalytic domain of the HSV polymerase, the UL30 protein contains an independent N-terminal 3′-to-5′ exonuclease domain (PolExo) that is important for proofreading during DNA replication. PolExo has been classified as a TMID enzyme based on structural homology with B-family polymerases (33, 37). In order to determine whether our panel of compounds could inhibit PolExo activity, a gel-based assay was used to detect degradation of a 25-nucleotide (nt) oligo(dT) substrate (Fig. 6A and B). Interestingly, BXA showed virtually complete inhibition of exonuclease activity at 20 μM. At this concentration, the other compounds had little or no effect on PolExo.

Although the polymerase domain of UL30 is not considered to be a TMID enzyme, DNA polymerases employ metal-coordinating acidic residues that are required for catalysis (62). Thus, it was important to determine the effect of these compounds on polymerase activity itself. A gel-based assay was used to monitor the ability of UL30 to extend a primed template. At a concentration of 20 μM, only AK-157 and the known polymerase inhibitor phosphonoacetic acid (PAA) showed strong inhibition of polymerase activity (Fig. 6B). At a significantly higher concentration (200 μM), AK-166 also inhibited HSV polymerase activity (data not shown).

The candidate-based approach revealed a number of compelling differences among the compounds in our panel. While the closely related 8-HQs AK-157 and AK-166 both inhibited the UL12 alkaline exonuclease, AK-157 also could inhibit UL30 polymerase activity. BXA showed strong inhibition of PolExo activity without significant effect on polymerase activity. It is noteworthy that this is the first report of a small molecule inhibitor of the proofreading exonuclease. Collectively, our results show that, despite the common metal-directed pharmacophore, the compounds presented in this study show unique profiles with respect to target inhibition.

### Effects of AK-157 and AK-166 on viral gene expression, viral DNA synthesis, and replication compartment formation.

**FIG 7:**
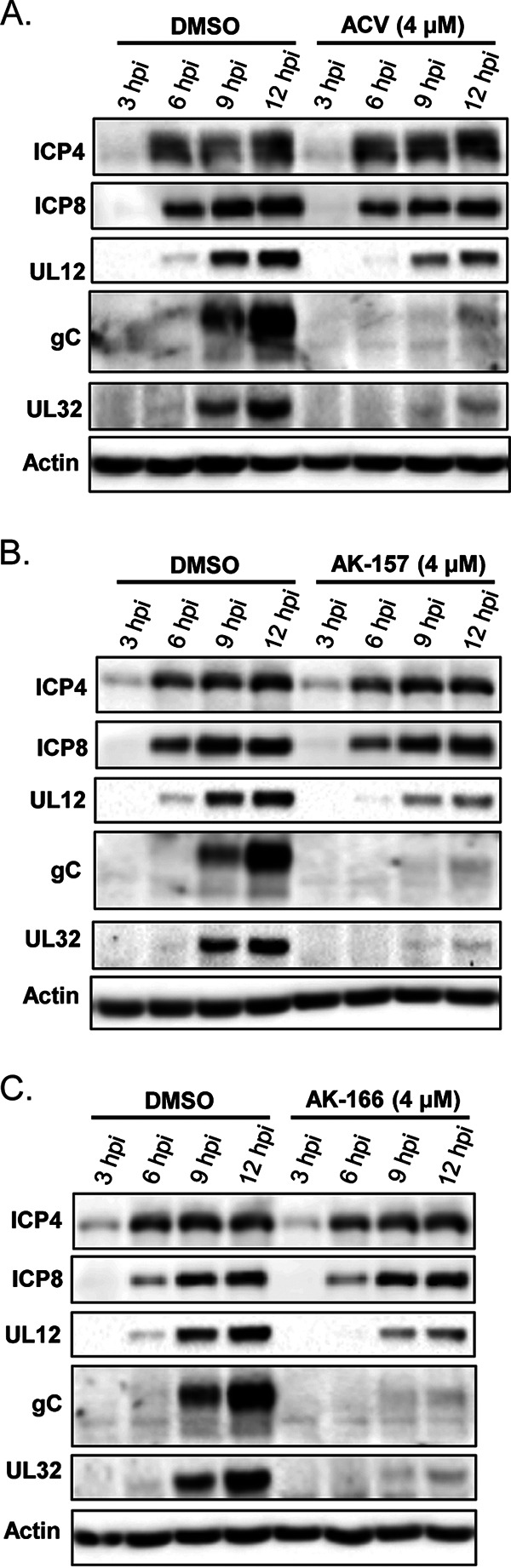
Viral gene expression. HFF cells were infected with KOS at an MOI of 5 PFU/cell in the presence of 4 μM compound ACV (A), AK-157 (B), or AK-166 (C) or DMSO alone for 3, 6, 9, and 12 h. Cell lysates were resolved by10% SDS-PAGE, transferred to PVDF membrane, and probed with antibodies to proteins representing all three kinetic classes of viral gene expression: (i) ICP4 (immediate early), (ii) ICP8 and UL12 (early), and (iii) gC and UL32 (late).

Of the original panel of compounds, the strongest correlation between antiviral properties and target-based inhibition was observed for the 8-HQs. To further investigate the potential mechanism of action of these agents, we evaluated their effects on the progression of viral infection by measuring gene expression, viral DNA synthesis, and replication compartment (RC) formation. HFF cells were infected at a multiplicity of infection (MOI) of 5 PFU/cell for 12 h at a 4.0 μM drug concentration. Viral gene expression in KOS-infected cells treated with AK-157 or AK-166 was monitored by Western blotting using antibodies to ICP4 (an immediate early [IE] protein), ICP8 and UL12 (early [E] proteins), and UL32 and gC (late [L] proteins) (Fig. 7). Cells treated with AK-157 and AK-166 displayed levels of IE and E proteins comparable to those seen in untreated cells; however, at 9 h postinfection (hpi), infected cells treated with either inhibitor exhibited decreased expression of the late proteins. The phenotypes observed with AK-157 and AK-166 are nearly identical to those seen with the ACV control, consistent with inhibition of viral DNA synthesis. To further confirm this result, we monitored viral DNA synthesis and replication compartment formation.

**FIG 8:**
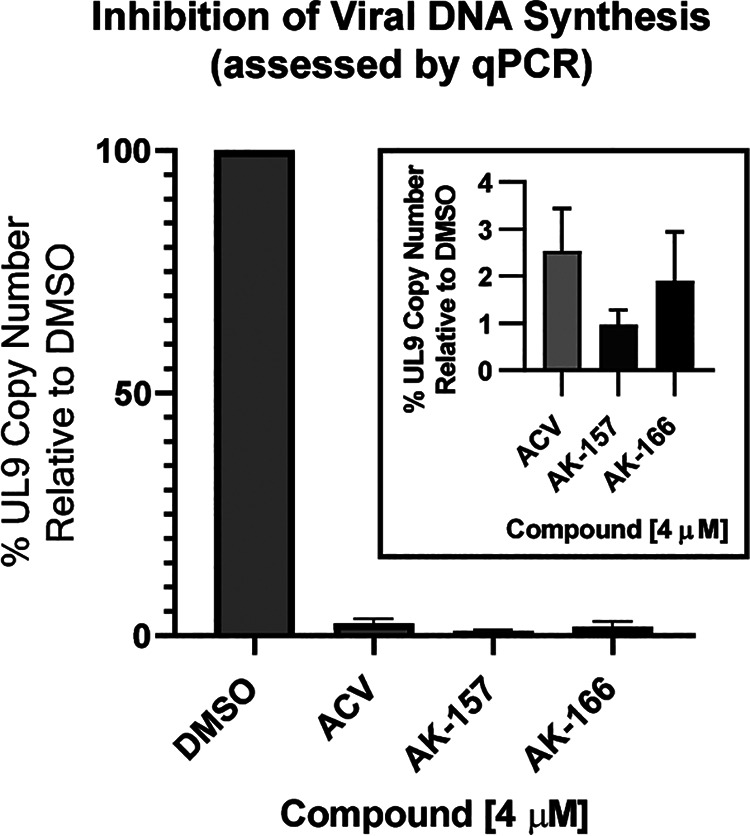
Viral DNA synthesis as assessed by qPCR. HFF cells were infected with KOS at an MOI of 5 PFU/cell and incubated in the presence of either compound or DMSO only for 12 h. qPCR was used to determine the amount of viral DNA by measuring the copy number of the UL9 gene. The accumulation of the 200-nt fragment from the UL9 gene was monitored using SsoAdvanced Universal SYBR green supermix (Bio-Rad). Data represent an average from three independent experiments.

**FIG 9:**
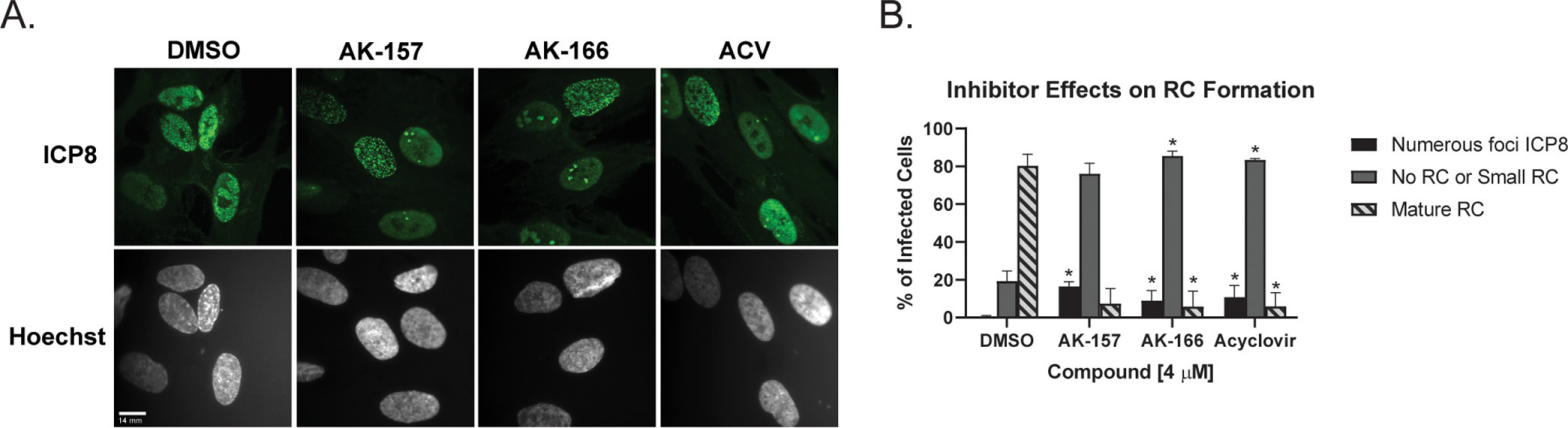
Replication compartment formation in the presence of compounds. (A) HFF cells were infected with KOS at an MOI of 5 PFU/cell in the presence of 0.5% DMSO or 4 μM AK-157 or ACV. At 6 hpi, cells were fixed for analysis by IF to detect ICP8 (green) or Hoechst staining to label nuclei (gray). (B) An average of 120 or more cells was counted from each treatment condition and classified according to the size of replication compartments. The prevalence of each category is presented as a percentage based on total ICP8-positive cells. Values represent an average from two independent experiments. *, *P* < 0.05 compared with the DMSO control by one-way ANOVA and Holm-Sidak multiple-comparison test.

Figure 8 shows viral genomic DNA replication as determined by quantitative PCR (qPCR) following treatment with AK-157, AK-166, or ACV at 4.0 μM for 12 h. All three compounds showed suppression of viral DNA synthesis compared to the DMSO-treated control. In previous work, we and others identified various stages in the formation of replication compartments (RCs) using ICP8 as a marker (64–67). The formation of RCs in infected cells requires active viral DNA synthesis (64, 68). HFF cells were treated with 4 μM AK-157, AK-166, or ACV for 6 h and stained with ICP8 antibodies to detect RCs. Figure 9A and B show that a majority of DMSO-treated cells exhibited large RCs that have grown to fill most of the nucleus, while the majority of AK-157-, AK-166-, and ACV-treated cells contained either no or small RCs. Taken together, these experiments indicate that 8-HQs block viral DNA synthesis.

Furthermore, cells treated with AK-157, AK-166, or ACV also exhibited a significant fraction of cells containing numerous small ICP8 foci (16, 8.9, and 10.7%, respectively). Interestingly, we have previously shown that the appearance of these numerous punctate foci is not a general result of inhibiting HSV DNA replication but rather requires specific inhibition of the viral polymerase (69). When viral DNA synthesis was blocked with a polymerase inhibitor (PAA or ACV), two types of ICP8 staining patterns were observed (70). One pattern is characterized by the formation of a limited number (<15) of small ICP8 foci that formed adjacent to promyelocytic leukemia protein (PML) nuclear bodies and eventually progressed into RCs if polymerase inhibition was reversed. On the other hand, cells that were in S-phase at the time of infection were shown to contain numerous (>20) smaller punctate ICP8 foci that costained with bromodeoxyridine (BrdU) and the cellular DNA damage response marker hyperphosphorylated RPA (65, 66, 69, 71). This numerous-staining pattern was not observed in cells infected with a polymerase null mutant (hp66), even in the presence of polymerase inhibitors, suggesting that the formation of numerous foci was dependent on the presence of an inactivated HSV polymerase (69). We thus proposed that in cells that are undergoing cellular DNA replication at the time of infection, the viral polymerase and ICP8 are recruited to sites of cellular DNA synthesis and that, if the viral polymerase is inhibited, replication stalls, inducing a DNA damage response and the hyperphosphorylation of RPA. Interestingly, treatment with a helicase-primase inhibitor (BAY 57-1293) (69) or BXA (data not shown) did not result in the induction of a DNA damage response. We therefore conclude that the induction of the cellular DNA damage response by 8-HQs provides further evidence that the compounds have a direct effect on viral DNA polymerase.

## DISCUSSION

The clinical success of integrase and PA inhibitors establishes that viral homologs of two-metal ion-dependent enzymes can effectively and selectively be targeted. In this article, we evaluated the inhibition of four putative HSV targets containing two-metal ion-dependent catalytic sites using a panel of molecules with metal-directed pharmacophores. This candidate-based approach was designed to assess the druggability of each target and correlate inhibition with antiviral activity: see Table 1 for a summary of the data presented. Several important observations were made. (i) Integrase inhibitors exhibited weak overall antiviral activity against HSV-1. (ii) Despite prior work claiming that the ssDNA binding protein (ICP8) was a potential target of raltegravir, the results described in this article did not support that hypothesis. (iii) An *in vitro* assay using the C-terminal domain of the HSV terminase (UL15C) failed to correlate target inhibition and antiviral activity, suggesting the need for alternative biochemical assays for this function. (iv) Compared with the integrase inhibitors, the anti-influenza agent BXA exhibited stronger antiviral activity, but also higher cytotoxicity. Biochemically, BXA inhibits the proofreading exonuclease of HSV polymerase (PolExo). (v) The more strongly antiviral 8-HQ compounds inhibited viral DNA synthesis, as assessed by gene expression, qPCR, and replication compartment formation. While both compounds effectively blocked the exonuclease activity of UL12, AK-157 also inhibited the catalytic activity of the HSV polymerase with much greater potency than AK-166. The interesting biochemical profile and antiviral activity of 8-HQs suggest the potential to develop dual-targeting agents for the inhibition of herpesviruses.

**TABLE 1:** Relative antiviral activities of the study compounds against HSV-1, juxtaposed with their relative biochemical activities against the targets of interest^*a*^

Compound	HSV-1 antiviral activity	Activity against target of interest				
		ICP8 (20 μM)	UL15C (12.5 μM)	UL12 (20 μM)	PolExo (20 μM)	Pol (20 μM)
RAL	−	−	+++	−	−	−
DTG	+	−	+++	−	−	−
MK2048	+	−	++	−	−	−
CTG	+	−	+++	−	−	−
XZ45	++++	−	+++	+	−	−
PPG	++	−	+++	++++	−	−
BTP	++++	−	+++	−	−	−
BXA	+++	−	+++	+	++++	−
AK-157	++++	−	+	++++	+	++++
AK-166	+++	−	++	++++	+	−

a The levels of inhibition at the indicated concentrations are presented as follows: −, no inhibition; +, weak inhibition; ++, moderate inhibition; +++, strong inhibition; ++++, complete inhibition.

### Repurposing of integrase inhibitors.

Previous reports have suggested that integrase inhibitors target ICP8 and UL15 and could be repurposed for treatment of HSV infections (23, 48, 49). Repurposing is an attractive strategy as it leverages considerable investments in drug development to rapidly address a different clinical need. In our hands, EC_50_ values against HSV-1 for all of the clinical integrase inhibitors exceeded 50 μM. This is in sharp contrast to the strong potency they exhibit against HIV—reported to be 1 to 2 nM for DTG and <8 nM for RAL (72, 73). Thus, it is unlikely that integrase inhibitors could be repurposed as anti-HSV agents, since it may prove very challenging to achieve sustainable serum concentrations above the EC_50_ value.

### Potential HSV targets of integrase inhibitors.

Previous evidence that ICP8 was a target for integrase inhibitors was interesting in light of our recent discovery that the annealing function of ICP8 is essential for DNA replication (60). However, we found that integrase inhibitors do not interfere with ssDNA binding or annealing, nor did RAL engage with ICP8 in a direct binding experiment. Although ICP8 has been reported to be a two-metal-binding protein, it does not bear obvious structural homology to either the RNHL or DEK TMID proteins, nor does it have an enzymatic nuclease activity typically associated with this large family of enzymes. We conclude that ICP8 does not appear to have a druggable metal-centered active site and is not likely to be a target of metal-directed pharmacophores. On the other hand, herpesvirus terminases are bona fide members of the RNHL superfamily of TMIDs. Consistent with previous reports (20, 21), we found that the integrase inhibitors and other compounds in our panel inhibited UL15C activity (Fig. 4). However, inhibition occurred at levels far lower than concentrations at which antiviral effects were observed. For example, RAL inhibited nearly 80% of the UL15C nuclease activity at 12.5 μM, while exhibiting almost no antiviral activity at a 4-fold-higher concentration. This evident disconnect calls into question the utility of this assay in discovering antiviral terminase inhibitors. Although the assay is convenient, robust, and scalable (21), it is important to note that it only includes the C-terminal fragment of UL15 in the absence of its known binding partners, UL28 and UL33. In addition, the assay does not reflect the sequence specificity of the terminase and does not truly mimic terminase function in infected cells. Thus, although terminase activity remains a promising target for antiviral drug discovery, our observation that inhibition of UL15C using this assay does not correlate with antiviral activity suggests that a different assay would be needed to better pursue such discovery efforts. Overall, the integrase inhibitors showed no evidence of activity against the other TMID targets, alkaline exonuclease and PolExo (Fig. 5 and 6). Although integrase inhibitors were designed to bind a similar two-metal-dependent site, the differences in potency toward the HSV TMIDs and integrase highlight the importance of structural features outside the metal-binding pharmacophore. The contribution of these additional interactions to potency and selectivity underscores the inherent difficulty in drug repurposing.

### Baloxavir inhibits HSV and CMV replication in cell culture and PolExo activity *in vitro*.

In contrast to the structurally similar integrase inhibitors, the anti-influenza agent BXA inhibits replication of HSV and CMV (EC_50_s of 9.96 and 4.29 μM, respectively) and inhibits PolExo at similar concentrations (Fig. 6). BXA is, unfortunately, associated with strong cytotoxicity, producing a very narrow selectivity index (SI). Thus, it is difficult to correlate the antiherpesvirus activity with a specific viral target and may suggest the involvement of cellular targets.

Although the polymerase function of UL30 has been exploited for the development of potent antivirals, BXA is the first antiviral agent ever reported to target the PolExo function. PolExo is important for proofreading during DNA replication (74–78); however, uncertainty remains about whether PolExo activity is essential for viral DNA synthesis (79–81). A validated inhibitor of PolExo function would provide an important tool for answering this question. In addition, inhibition of PolExo may produce a high degree of instability in progeny genomes by increasing the mutational burden. Further studies on the effects on BXA on PolExo and polymerase activity and replication fidelity are ongoing.

### Tropolone and catechol derivatives XZ45, BTP, and PPG.

We evaluated three compounds containing metal binding catechol (XZ45), tropolone (BTP), or both moieties (PPG). Although PPG had relatively weak antiviral activity, it showed a high degree of inhibition against UL15C and UL12. At higher concentrations, PPG also showed activity against ICP8, PolExo, and Pol. PPG is a natural product that has been associated with a wide range of biological activities, including interaction with several DNA binding proteins (61). While the results with PPG are interesting, it is important to note that the compound exhibits exceptionally strong metal-chelating ability, which could produce a wide variety of pharmacological effects. XZ45 was originally investigated as an integrase inhibitor; however, the presence of catechol and acylhydrazide functionalities in this compound raises concerns about its drug-like properties. BTP and XZ45 exhibited strong HSV activity, consistent with previous reports (25, 48); however, the target-based evaluation revealed little or no inhibition of ICP8, UL12, or either of the polymerase functions (PolExo or Pol). We cannot rule out UL15 terminase or other viral or cellular targets that may mediate the observed antiviral effects of these two compounds.

### 8-HQs are antiviral and may be dual targeting.

We have created a focused library of small molecules to target TMIDs and have identified two 8-HQs (AK-157 and AK-166) with good anti-HSV activity (EC_50_s of 0.17 and 0.98 μM, respectively). Other than the pleotropic PPG, only AK-157 and AK-166 showed potent activity against UL12 alkaline nuclease (Fig. 5). AK-157 was also shown to be a very strong polymerase inhibitor (Fig. 6B), while AK-166 only showed polymerase inhibition at significantly higher concentrations. The observation that AK-157 inhibits both UL12 and UL30 polymerase activities with high potency is intriguing and suggests that it may be possible to develop a dual-targeting agent.

The inhibition of two or more essential enzymes with a single agent would be expected to generate a synergy in which the combined effects of a dual-targeting agent might exceed the additive effect of inhibiting one at a time. A multitargeting agent would be expected to reduce resistance and allow treatment at significantly lower doses. It is noteworthy that AK-157 and AK-166 share identical metal-binding pharmacophores and differ only in the composition of the amide side chain, demonstrating that small structural changes outside the metal-binding region can have a large impact on target engagement.

### Summary.

Overall, these studies identify several potential TMID enzymes encoded by HSV-1 that could be targeted by small molecule inhibitors. The inhibition of PolExo by BXA adds a potential new candidate for further exploration. We also report that AK-157 inhibits both UL12 and Pol activities. These results raise the exciting possibility of development of one agent that targets two different viral proteins. Furthermore, the results presented in this article indicate that several metal-binding compounds exhibit antiviral activity against both HSV and CMV, consistent with broad-spectrum inhibition. This result is supported by the observation that the active sites of several TMID proteins, including alkaline nuclease, terminase, and polymerase, are well conserved across HHVs.

## MATERIALS AND METHODS

### Cells and viruses.

African green monkey kidney cells (Vero) were obtained from ATCC and propagated as described previously (82). Limited-passage primary human foreskin fibroblast (HFF) cells were purchased from ATCC and cultured in minimum essential media (MEM) (Invitrogen, Carlsbad, CA) supplemented with 10% fetal bovine serum (FBS) and 0.1% penicillin–streptomycin. For baculovirus protein expression, Sf9 insect cells were obtained from Thermo Fisher Scientific (Gibco) and maintained in suspension culture using Sf-900 II serum-free medium (SFM). The wild-type KOS strain of HSV-1 was used in HSV inhibition assays. The pp28-luciferase-expressing HCMV Towne strain was used in CMV assays and was generously provided by Donald Coen (Harvard University). pp28-luciferase HCMV expresses luciferase under the control of the pp28 late promoter and was constructed as described previously (83).

### Compounds.

RAL, MK-2048, and CAB were purchased from 1 Click Chemistry (Kendall Park, NJ), and DTG was purchased from AK Scientific (Union City, CA). BXA was obtained from Sigma-Aldrich (St. Louis MO). XZ-45, AK-157, and AK-166 were synthesized at the University of Connecticut (see supplemental data for compound characterization). Compounds were dissolved in 100% DMSO at a concentration of 50 mM and stored at −20°C.

### Antiviral experiments.

HFF cells were seeded in 96-well plates in 5% FBS–MEM. At 24 h postseeding, cells were infected with KOS at a multiplicity of infection (MOI) of 0.1 PFU/cell or pp28-luciferase-expressing virus at an MOI of 2 PFU/cell and incubated for 48 or 65 h, respectively, in the presence of various concentrations of the compounds or DMSO alone in 100 μL MEM at final concentrations of 2% FBS and 0.5% DMSO. For the HSV inhibition assay, the entire plate was subjected to 2 freeze-thaw cycles, and viral yields were assessed by plaque assay on Vero cells as described previously (82). To measure CMV inhibition, cells were monitored for luciferase expression using the SteadyGlo luciferase assay kit (Promega, Madison WI) according to the manufacturer’s instructions.

### Cytotoxicity assay.

HFF cells were seeded in 96-well plates in 5% FBS–MEM. At 24-h postseeding, cells were incubated in the presence of various concentrations of the compounds or DMSO alone in 100 μL of MEM at final concentrations of 2% FBS and 0.5% DMSO. At 65 h posttreatment, cytotoxicity was assessed using CellTiter Glo (Promega, Madison WI) luciferase assay according to the manufacturer’s instructions.

### Protein expression and purification.

ICP8 (84–86) and UL12 (16) were purified from baculovirus-infected Sf9 insect cells as described previously. The HisUL30 expression clone was constructed by amplification of the UL30 gene by PCR using primers containing an N-terminal 6×His tag and a HindIII restriction site. The construct was inserted into the HindIII site of the pFastBac plasmid. His-tagged UL30 was purified as previously described (87, 88). His-UL30ΔN42 protein (89) was a generous gift from Donald Coen (Harvard Medical School). Recombinant UL15C (residues 471 to 736) was constructed by amplifying the C-terminal domain of UL15 followed by insertion between the NdeI and XhoI restriction sites of pET28b(+). Recombinant UL15C was purified from Escherichia coli BL21(DE3) cells as previously described (35).

### PicoGreen alkaline nuclease assay.

Alkaline exonuclease (UL12) activity was monitored by the PicoGreen assay as described previously (16, 63). Briefly, 25-μL reaction mixtures containing 20 mM Tris-HCl (pH 8.2), 40 mM NaCl, 1 mM MgCl_2_, 1 mM dithiothreitol (DTT), 10 nM UL12, and 1.6% DMSO or 20 μM inhibitor in 100% DMSO were preincubated at 37°C for 10 min prior to addition of DNA substrate. The nuclease reaction was initiated by adding 1 nM linearized pUC19 DNA, the mixture was incubated at 37°C for an additional 5 min, and the reaction was quenched by the addition of 10 μL 2.5 mM EDTA (pH 8.0). The quenched reactions were processed for PicoGreen fluorescence according to the manufacturer’s protocol (Invitrogen). Briefly, each quenched reaction was diluted to 100 μL by the addition of Tris-EDTA (TE [pH 7.5]), mixed with 100 μL of PicoGreen (1:200 diluted in TE buffer), and transferred to a 96-well Flurotrac 200 black plate (Greiner Bio-One). Fluorescence was measured using a SpectraMax 3 plate reader with excitation and emission wavelengths of 480 and 520 nm, respectively.

### UL15C dual-probe fluorescence nuclease assay.

The dual-probe fluorescence assay was performed as previously described (21). Briefly, the dually labeled oligonucleotide substrate was denatured and reannealed by heating a mixture containing 10 μM substrate, 10 mM Tris-HCl (pH 7.6), and 25 mM NaCl to 80°C and allowed to slowly cool to 4°C. To assess nuclease activity, in a 96-well Flurotrac 200 black plate (Greiner Bio-One), 100-μL reaction mixtures containing 20 mM Tris-HCl (pH 7.0), 10 mM NaCl, 1 mM MnCl_2_, 250 nM UL15C, and 1% DMSO or 12.5 μM inhibitor (in 100% DMSO) were preincubated at 37°C for 10 min in a SpectraMax 3 plate reader before the addition of the DNA substrate. Reactions were initiated by adding DNA duplex to a final concentration of 250 nM, the mixture was incubated for an additional 10 min at 37°C, and fluorescence was measured at emission and excitation wavelengths of 646 and 670 nm, respectively. Wells containing DMSO were used as controls. The background fluorescence due to inhibitors was subtracted from individual measurements, and the percentage of inhibition was calculated based on relative fluorescence units (RFU) as 100× (RFU_DMSO_ − RFU_inhibitor_)/RFU_DMSO_.

### UL30 polymerase assay.

The UL30 polymerase assay was conducted using His-tagged UL30ΔN42. The primed substrate contained a 5′-FAM (6-carboxyfluorescein)-labeled 15-mer annealed to a 35-mer oligonucleotide. Each reaction mixture contained 50 nM HisUL30ΔN42, 50 mM HEPES (pH 7.6), 2 mM DTT, 10 mM MgCl_2_, 60 mM deoxynucleoside triphosphates (dNTPs), and the indicated concentrations of inhibitors. Reaction mixtures were assembled on ice and preincubated for 10 min at 37°C to ensure formation of the inhibitor-protein complex. The reactions were initiated by adding the primer-template substrate at a final concentration of 100 nM, the mixtures were incubated at 37°C for an additional 5 min, and the reactions were quenched by the addition of 2 volumes of 2× loading dye containing 90% formamide, 10 mM EDTA, 1× Tris-borate-EDTA (TBE) buffer, and 0.01% bromophenol blue. Samples were heated for 3 min at 75°C and snap-cooled on ice for 5 min, and the reaction mixtures were separated on a 20% denaturing (7.5 M urea) polyacrylamide gel. FAM-labeled substrate was detected at 520 nm following excitation at 495 nm using a ChemiDoc MP imaging system (Bio-Rad).

### UL30 exonuclease (PolExo) assay.

PolExo was monitored in the absence of dNTPs using purified His-tagged UL30. A 5′-Cy5-labeled dT 25-mer was used as the substate for the exonuclease reactions. Reaction mixtures containing final concentrations of 100 nM His-UL30, 50 mM HEPES (pH 7.6), 2 mM DTT, and 10 mM MgCl_2_ were assembled on ice with various concentrations of inhibitors. Polymerase and inhibitor-containing reaction mixtures were preincubated at 37°C for 10 min prior to addition of the substrate. The exonuclease activity was initiated by adding the 5′-Cy5-labeled dT 25-mer substrate (100 nM final concentration), the mixture was further incubated for 8 min at 37°C, and the reaction was quenched with addition of 2 volumes of 2× loading dye containing 90% formamide, 10 mM EDTA, 1× Tris-borate-EDTA buffer, and 0.01% bromophenol blue. Samples were processed under the polymerase assay conditions and separated on a 20% denaturing (7.5 M urea) polyacrylamide gel, and Cy5-labeled substrate was detected at 650 nm by excitation at 671 nm using ChemiDoc MP imaging system (Bio-Rad).

### ICP8 annealing of complementary ssDNA.

ICP8 at a concentration of 100 nM was preincubated with either DMSO or compound (20 μM or 100 μM with a 1.6% DMSO final concentration) for 10 min at 37°C. Annealing was initiated by the addition of 50 ng linearized, heat-denatured plasmid DNA (pSAK), and the mixture was incubated at 37°C for 45 min. Samples were collected at 0 min and 45 min, and the production of dsDNA was assessed by PicoGreen fluorescence (60). The data presented represent the average from three independent experiments. Error bars represent the standard deviation.

### ICP8 ssDNA binding assay (EMSA).

ICP8 (200 nM) was preincubated with various concentrations of compound (25 to 400 μM) or DMSO (the final DMSO concentration across all conditions was limited to 2%) for 10 min at 37°C. Reactions were initiated by the addition of ssDNA oligonucleotide (Cy5-labeled dT 25-mer; IDT) to a final concentration of 100 nM in a DNA binding buffer (20 mM Tris-HCl [pH 7.5], 4% glycerol, 0.1 mg/mL bovine serum albumin [BSA], 0.5 mM DTT, 5 mM MgCl_2_). Samples were incubated for 30 min at room temperature and quenched with 40% sucrose solution. Bound and unbound DNA species were separated on 5% nondenaturing polyacrylamide gel in 1× Tris-borate-EDTA (TBE) and imaged using the ChemiDoc MP imaging system (Bio-Rad).

### Thermal shift assay.

Samples were prepared by combining equal volumes of purified ICP8Δ60 (3.5 μM in HEPES [pH 7.5], 100 mM NaCl, 0.1 mM EDTA, 15% glycerol, 2.5% DMSO, 5 mM MgCl_2_, and 2 mM TCEP) with the same buffer containing or not containing ssDNA (7 μM for a 2× molar excess to protein) and the drug (70 μM for a 20× molar excess to protein). The samples were incubated overnight at 4°C to ensure complex formation and loaded (10 μL) into a capillary. Capillaries were subjected to increasing temperatures from 35°C to 95°C at a rate of 30°C/min. Changes in intrinsic fluorescence intensity from tryptophan residues were used to calculate derivatives of the signal (ratio of fluorescence at 350 nm to that at 330 nm) using the evaluation features provided by the Tycho NT.6 instrument (Nanotemper, Munich, Germany). The maximum of the peak corresponds to the melting temperature of the protein (inflection point of the underlying ratio curve [*T_i_*]) and serves as a marker of protein structural integrity.

### Western blot analysis.

HFF cells seeded onto a 24-well plate were infected with KOS at an MOI of 5 PFU/cell and incubated in the presence of compounds or DMSO in 500 μL MEM with final concentrations of 2% FBS and 0.5% DMSO. At the indicated times, cells were lysed in 1.5× SDS buffer (3% SDS, 15% glycerol, 75 mM Tris [pH 6.8], 150 mM DTT, and 0.1% bromophenol blue), heated at 95°C for 5 min, and stored at −20°C. Proteins in cell lysates were separated by SDS-PAGE and transferred to polyvinylidene difluoride (PVDF) membranes. Membranes were blocked with 5% milk and probed with antibodies: mouse monoclonal anti-ICP4 (1:1,000; Santa Cruz no. sc69809), rabbit polyclonal anti-ICP8 (1:5,000; clone 3-83, a gift from David Knipe, Harvard Medical School), rabbit polyclonal anti-UL12 antibody (1:10,000; a gift from Joel Bronstein and Peter Weber, Parke-Davis Pharmaceutical Research), mouse monoclonal anti-gC (1:1,000; Abcam no. ab6509), rabbit polyclonal anti-UL32 (1:500; antibody to synthetic antigenic peptide was generated by Open Biosystems), and mouse antiactin antibody (1:10,000; Sigma catalog no. A5441).

### qPCR measurements of viral DNA synthesis.

HFF cells seeded onto a 24-well plate were infected with KOS at an MOI of 5 PFU/cell and incubated in the presence of compounds or DMSO in 500 μL MEM with final concentrations of 2% FBS and 0.5% DMSO. Samples were collected at 12 hpi, and total DNA was isolated using QIAamp DNA blood minikit (Qiagen) according to the manufacturer’s instructions. Analysis of viral DNA replication using real-time qPCR was conducted as described previously (16) using primers corresponding to the coding sequence of the UL9 gene. Accumulation of DNA products was measured using SsoAdvanced Universal SYBR green.

### Immunofluorescence.

HFF cells cultured on glass slides in a 24-well plate were infected with KOS at an MOI of 5 PFU/cell and incubated in the presence of compounds or DMSO in 500 μL MEM at concentrations of 2% FBS and 0.5% DMSO. At 6 hpi, cells were processed for immunofluorescence (IF) analysis as described previously (90). Imaging was performed using a 63× Plan Apochromat lens objective. Protein levels were detected using polyclonal rabbit anti-ICP8 antibody (clone 367 at 1:1,000; a gift from William Ruyechan) and Alexa Fluor secondary antibody (Molecular Probes) conjugated with a fluorophore excitable at 488 nm. Cell nuclei were detected with Hoechst stain (gray).

### Statistical methods.

For all outcomes that were statistically analyzed, means of sample replicates for each compound (*n* = 2 to 3/experiment) and the DMSO control (*n* = 3 to 6/experiment) from three independent experiments were analyzed. Most treatment groups were compared with the DMSO control using a one-way analysis of variance (ANOVA) and the Holm-Sidak multiple-comparison test. HFF assays in Fig. 2 were analyzed by two-way ANOVA (using compound and concentrations as factors) and the Holm-Sidak multiple-comparison test. A *P* value of < 0.05 was considered significant. Statistics were performed using GraphPad Prism (version 9.3). For figure graphs, significance is indicated by asterisks based on the raw data comparisons described above.

10.1128/mbio.03226-21.2 FIG S2 Label-free differential scanning fluorimetry (nanoDFS) of raltegravir and purpurogallin in complex with ICP8Δ60 with and without DNA. (A) The first derivative of the ratio of fluorescence at 350 nm to that at 330 nm (ΔF350nm/ΔF330nm) as a function of temperature. (B) The fluorescence ratio (F350nm/F330nm) as a function of temperature. Points represent *T_i_* in the presence of different binding partner(s). (C) Inflection temperatures for DSF experiments. Data are from panel A. Download FIG S2, PDF file, 0.3 MB. Copyright © 2022 DiScipio et al. 2022 DiScipio et al. https://creativecommons.org/licenses/by/4.0/ This content is distributed under the terms of the Creative Commons Attribution 4.0 International license.
