# Supplementary material for: Two-Metal Ion-Dependent Enzymes as Potential Antiviral Targets in Human Herpesviruses
Source: mBio. 2022 Jan 25;13(1):e03226-21. doi: 10.1128/mbio.03226-21 (PMC8787488; doi:10.1128/mbio.03226-21)
Supplement: FIG S1 [file mbio.03226-21-sf001.pdf]

Characterization data for compounds AK-157, AK-166 and XZ45 synthesized in this study

***N*-(4-bromophenyl)-8-hydroxyquinoline-7-carboxamide(AK-157):** Recrystallized from EtOAc, pinkish solid (42%); R_f value 0.68 (EtOAc/MeOH = 90:10); mp 203-204 °C; IR (neat) ν max: 3325, 3262, 1649, 1584, 1511, 1432, 1389, 1304, 1236, 1191, 1124, 1070, 1008, 949 cm^{-1}; ^1H NMR (DMSO-d_6, 500 MHz) δ (ppm) 11.17 (s, 1H), 8.93 (dd, *J* = 4.4, 1.6 Hz, 1H), 8.50 (dd, *J* = 8.3, 1.6 Hz, 1H), 8.01 (d, *J* = 8.7 Hz, 1H), 7.77 – 7.69 (m, 3H), 7.56 (d, *J* = 8.8 Hz, 2H), 7.41 (d, *J* = 8.7 Hz, 1H); ^{13}C NMR (DMSO-d_6, 125 MHz) δ (ppm) 165.9, 156.0, 147.9, 138.7, 138.3, 132.2, 131.3, 127.6, 123.9, 122.5, 116.1, 115.9, 115.8; HRMS (ESI) (m/z) calcd for $[C_{16}H_{11}BrN_2O_2 + H]^+$, 343.0071, observed 343.0042

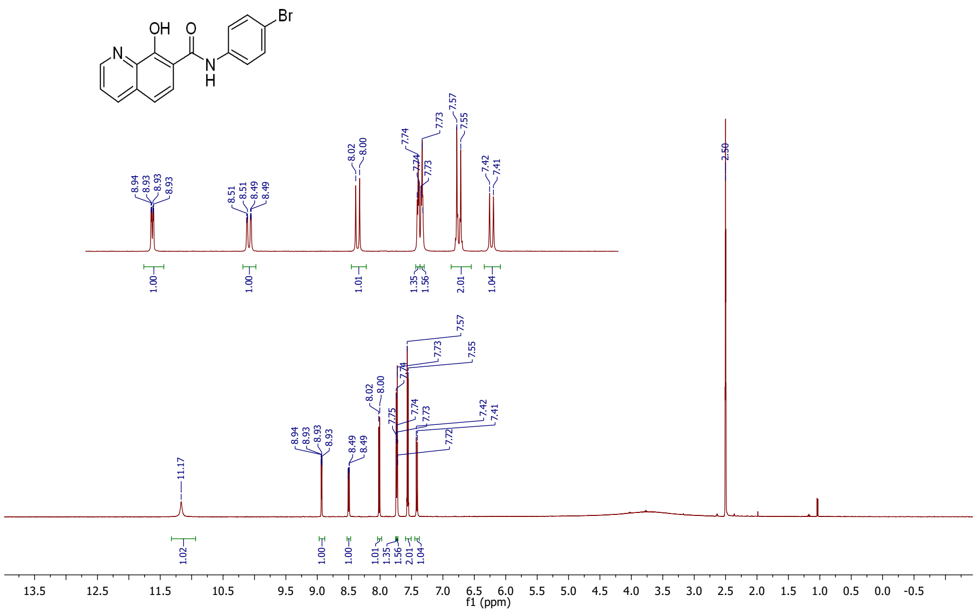

Fig S1a. ^1H NMR spectra for AK-157

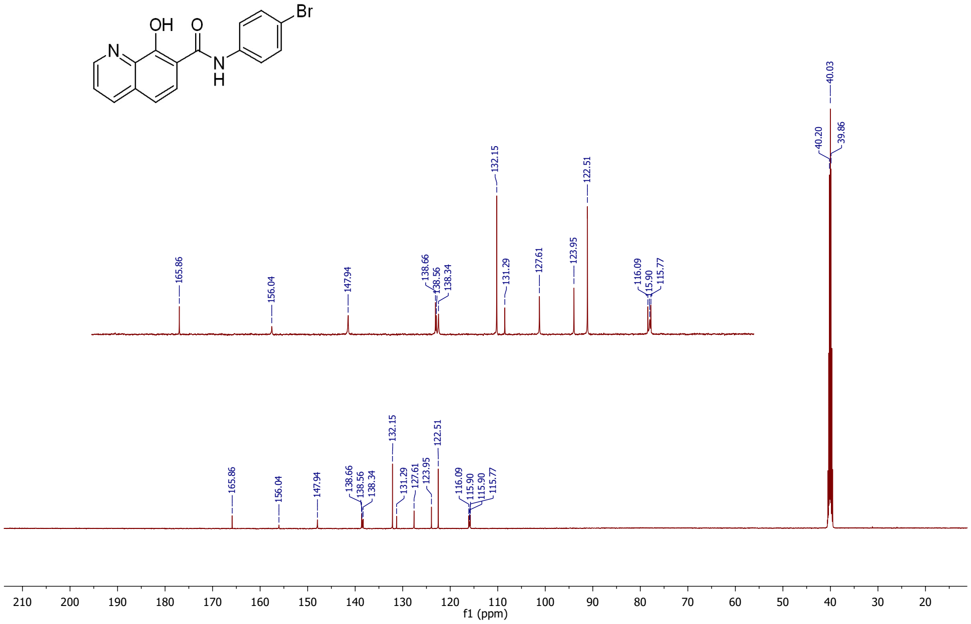

Fig S1b. ^{13}C NMR spectra for AK-157

8-hydroxy-*N*-(4-isopropylbenzyl)quinoline-7-carboxamide(AK-166):

Recrystallized from isopropanol, white solid (19%); R_f value 0.40 (EtOAc/MeOH = 90:10); mp 149.5-155.5 °C; IR (neat) ν max: 3371, 3290, 1639, 1531, 1468, 1433, 1386, 1330, 1294, 1199, 1159, 1097, 1045, 834, 663 cm^{-1}; ^{1}H NMR (DMSO-d_6, 500 MHz) δ (ppm) 9.30 (t, J = 6.0 Hz, 1H), 8.91 (dd, J = 4.2, 1.6 Hz, 1H), 8.35 (dd, J = 8.3, 1.6 Hz, 1H), 8.02 (d, J = 8.8 Hz, 1H), 7.65 (dd, J = 8.3, 4.2 Hz, 1H), 7.43 (d, J = 8.8 Hz, 1H), 7.29 (d, J = 8.0 Hz, 2H), 7.22 (d, J = 8.0 Hz, 2H), 4.55 (d, J = 5.9 Hz, 2H), 1.19 (d, J = 6.8 Hz, 6H); ^{13}C NMR (DMSO-d_6, 125 MHz) δ (ppm) 168.4, 157.2, 149.6, 147.6, 136.9, 136.5, 131.2, 128.0, 126.8, 125.6, 124.0, 117.4, 113.1, 42.9, 33.6, 24.4; HRMS (ESI) (m/z) calcd for $[C_{20}H_{20}N_2O_2 + H]^+$, 321.1592, observed 321.1635.

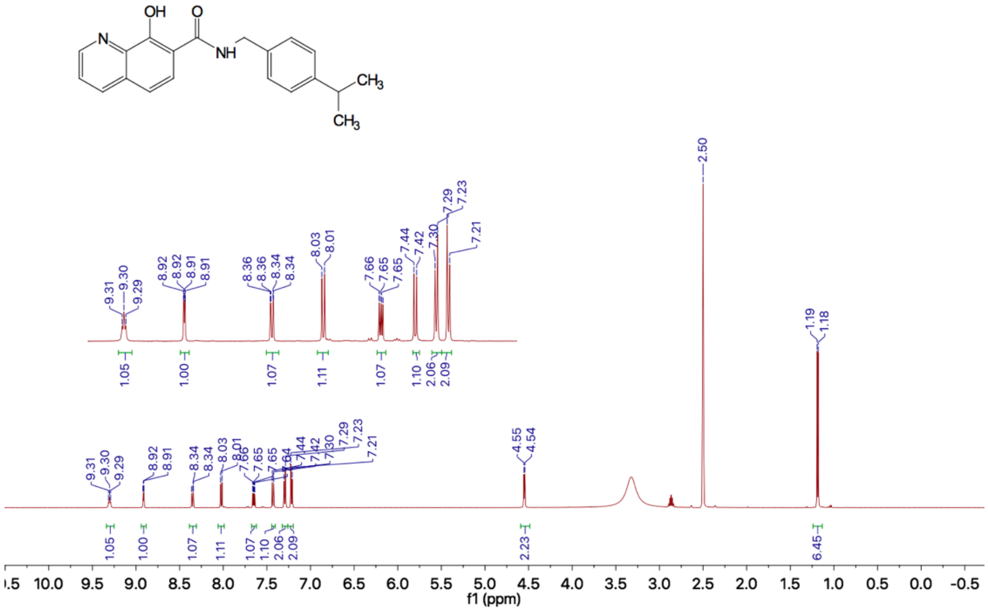

Fig S1c. ^{1}H NMR spectra for AK-166

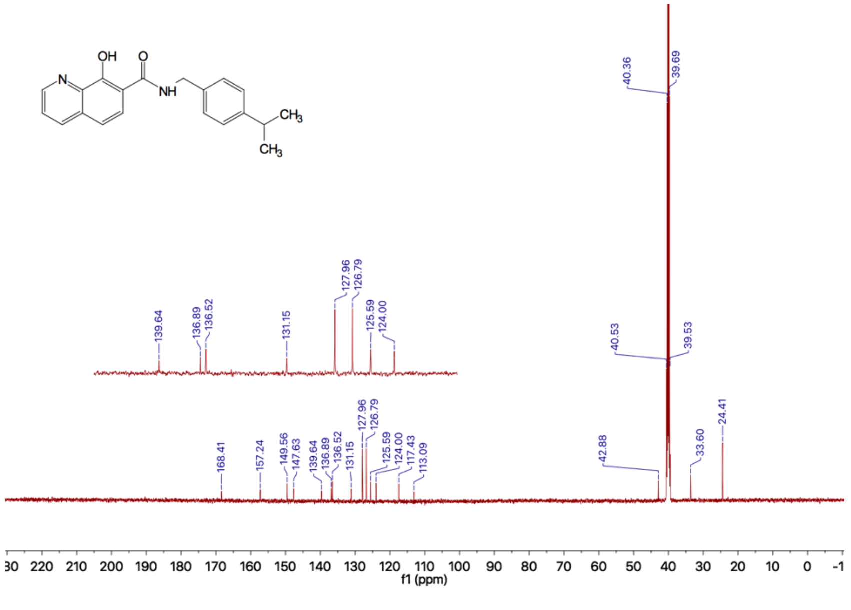

Fig S1d. ^{13}C NMR spectra for AK-166

XZ-45: ^{1}H NMR (DMSO) δ 11.74 (bs, 1H), 11.59 (bs, 1H), 10.83 (bs, 1H), 10.76 (bs, 1H), 7.87 (dd, 1H, J = 1.6 Hz, 8.0 Hz), 7.41 (dt, 1H, J = 1.6 Hz, 7.2 Hz), 7.33 6.96-6.90 (dd, 1H, J = 1.6 Hz, 8.0 Hz), 6.96-6.90 (m, 3H), 6.72 (t, 1H, J = 8.0 Hz); ^{13}C NMR (DMSO) δ 167.7, 166.8, 159.1, 148.9, 146.6, 134.6, 129.1, 119.7, 119.6, 119.1, 118.4, 117.7, 115.3, 114.9; FAB-MS m/z 287.1 (M-H). HRMS calcd for $C_{14}H_{13}N_2O_5$ [MH+]: 289.0824. Found: 289.0829.
