# Supplementary material for: Two-Metal Ion-Dependent Enzymes as Potential Antiviral Targets in Human Herpesviruses
Source: mBio. 2022 Jan 25;13(1):e03226-21. doi: 10.1128/mbio.03226-21 (PMC8787488; doi:10.1128/mbio.03226-21)
Supplement: FIG S2 [file mbio.03226-21-sf002.pdf]

A.

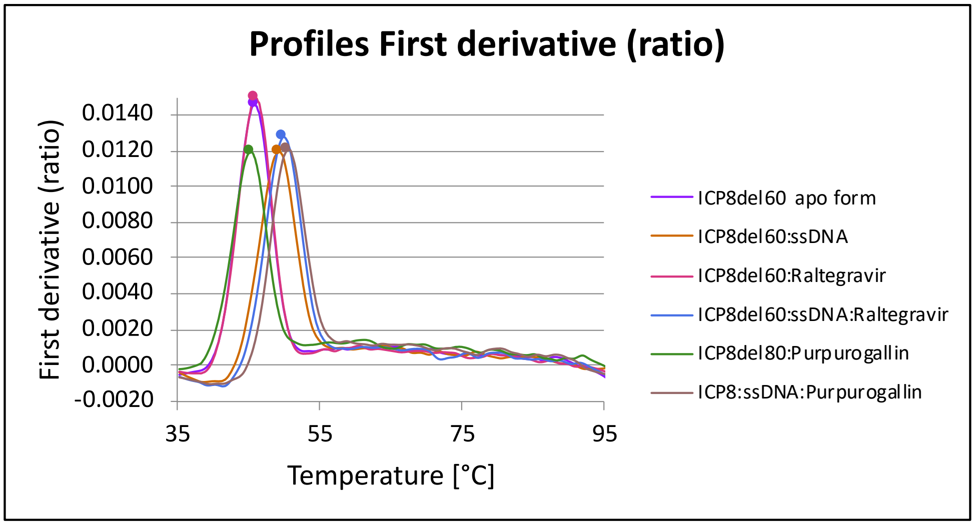

B.

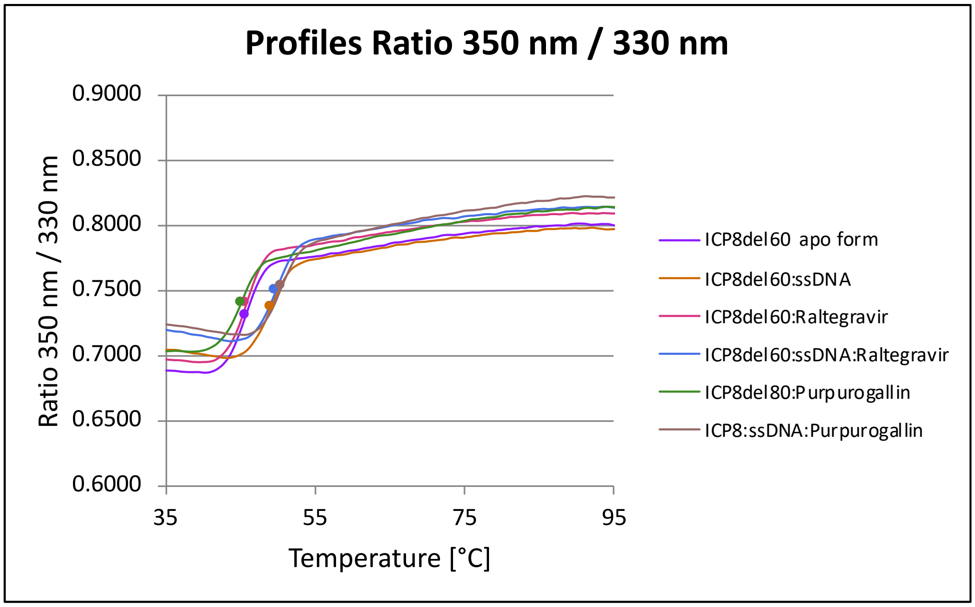

C.

Inflection Temperatures upon ligand binding		
**Sample ID**	**T_i [°C]**	**ΔT_i [°C] between ICP8del60 apo form and ICP8del60:ssDNA:+/- ligand**
ICP8del60 apo form	45.7	
ICPdel60:ssDNA	49.2	3.5
ICP8del60:Raltegravir	45.8	0.1
ICP8del60:ssDNA:Raltegravir	49.8	3.5/0.6
ICP8del60:Purpurogallin	45.1	-0.6
ICP8del60:ssDNA:Purpurogallin	50.5	3.5/1.3

FIG S2

Label-free Differential Scanning Fluorimetry (nanoDFS) of Raltegravir and Purpurogallin in complex with ICP8∆60 with and without DNA. A) The first derivative of the fluorescence ratio (∆*F*350nm/∆*F*330nm) as a function of temperature. B) The fluorescence ratio (*F*350nm/*F*330nm) as a function of temperature. Points represent T_i in the presence of different binding partner(s). C) Inflection temperatures for DSF Experiments. Data from Panel A.
